# Olfaction in the Multisensory Processing of Faces: A Narrative Review of the Influence of Human Body Odors

**DOI:** 10.3389/fpsyg.2021.750944

**Published:** 2021-10-05

**Authors:** Fabrice Damon, Nawel Mezrai, Logan Magnier, Arnaud Leleu, Karine Durand, Benoist Schaal

**Affiliations:** Developmental Ethology and Cognitive Psychology Laboratory, Centre des Sciences du Goût et de l’Alimentation, Inrae, AgroSup Dijon, CNRS (UMR 6265), Université Bourgogne Franche-Comté, Dijon, France

**Keywords:** multisensory perception, olfaction, vision, body odor, face processing, emotion, adults, infants

## Abstract

A recent body of research has emerged regarding the interactions between olfaction and other sensory channels to process social information. The current review examines the influence of body odors on face perception, a core component of human social cognition. First, we review studies reporting how body odors interact with the perception of invariant facial information (i.e., identity, sex, attractiveness, trustworthiness, and dominance). Although we mainly focus on the influence of body odors based on axillary odor, we also review findings about specific steroids present in axillary sweat (i.e., androstenone, androstenol, androstadienone, and estratetraenol). We next survey the literature showing body odor influences on the perception of transient face properties, notably in discussing the role of body odors in facilitating or hindering the perception of emotional facial expression, in relation to competing frameworks of emotions. Finally, we discuss the developmental origins of these olfaction-to-vision influences, as an emerging literature indicates that odor cues strongly influence face perception in infants. Body odors with a high social relevance such as the odor emanating from the mother have a widespread influence on various aspects of face perception in infancy, including categorization of faces among other objects, face scanning behavior, or facial expression perception. We conclude by suggesting that the weight of olfaction might be especially strong in infancy, shaping social perception, especially in slow-maturing senses such as vision, and that this early tutoring function of olfaction spans all developmental stages to disambiguate a complex social environment by conveying key information for social interactions until adulthood.

## Introduction

Humans have long been considered by academia as “microsmatic” mammals, with the underlying idea that the sense of smell plays only a minor role in human behavior and cognition. This flawed conception may be traced back to the 19th-Century writings of the comparative neuroanatomist Paul Broca (notably Broca, 1888; Schaal and Porter, 1991; cf. McGann, 2017). But Broca had illustrious contemporaries who shared similar ideas [e.g., Darwin who affirmed that, to Man, “the sense of smell is of extremely slight service, if any” (Darwin, 1871/1992)], and numerous were the followers who forced this line of reasoning in propagating an explicit conceptual glide from Broca’s initial structural observations into unfounded functional inferences (e.g., Freud, 1930/1962). This “microsmaty fallacy” (Lundström and Olsson, 2010) long pervaded scientific psychology for a variety of reasons (Sela and Sobel, 2010; McGann, 2017), including the consideration that the human vomeronasal organ is vestigial (Doty, 2001; Stowers and Spehr, 2015), the yet unsuccessful search for human pheromones (Doty, 2010; Wyatt, 2015, 2017), and the fact that humans had no obvious scent glands (Stoddart, 1990; Schaal and Porter, 1991). Recently, however, a growing amount of studies in biology, psychology, and anthropology stressed the overlooked role of olfaction in human behavior, and in particular in social cognition (e.g., Stoddart, 1990; Schaal and Porter, 1991; Classen et al., 2002; Stevenson, 2010; de Groot et al., 2017; Pause, 2017). As recent reviews have excellently covered the effects on face perception of arbitrary odors selected for their hedonic valence (Syrjänen et al., 2021, see also Spence, 2021), the present essay will survey investigations on how *natural human body odors*, or isolated compounds therein, can modulate the perception and emotional appraisal of faces. Our analysis of the current literature will exploit the lens of face processing under various olfactory influences in following evolutionary and developmental perspectives. Note that the current review is narrative and includes various papers selected without systematic methodology. However, the target articles included in the review (i.e., those involving specifically body odors influence on face perception) have been selected from a screening of databases (PubMed, Medline, and Web of Science) and the use of search engines (Google Scholar, Google) using the following keywords and their combinations: “body odor,” “face,” “multisensory,” “infants.” Papers related to non-human populations, and/or artificial odors were not included. Studies cited in the selected papers were also reviewed, and included whenever relevant to the topic.

## The Social Nose

Both face and voice convey a wealth of visual and acoustic information upon which humans rely ubiquitously to organize their everyday social interactions (e.g., Latinus and Belin, 2011; Quinn and Macrae, 2011; Pascalis et al., 2014; Schirmer and Adolphs, 2017). But, aside from vision and audition, olfaction increasingly appears as another effective means to sample social information from conspecifics, especially through the perception of volatile compounds emitted from multiple bodily sites (Schaal and Porter, 1991; Havlicek and Roberts, 2009; Pause, 2012; Lübke and Pause, 2015; de Groot et al., 2017). Olfactory communication is advantageous for several reasons (Wyatt, 2003). First, it can function when vision and audition are restricted or absent (e.g., in noisy or dark environment: Doty, 1986; in privation of vision: e.g., Cuevas et al., 2009). Second, odorants disperse in space and persist over time, allowing to communicate about past events (e.g., long after the sender’s departure can low volatile molecules inform about his/her state at the place of release; Pause, 2012), and making odors good candidates to support social communication (Wysocki and Preti, 2004; Doty, 2010; de Groot et al., 2017). However, an apparent pitfall of olfaction is that odor experience is notoriously hard to translate into words (Engen, 1987; Yeshurun and Sobel, 2010), which undermines the usefulness of self-reports. This led some researchers to consider olfaction as a “muted sense” (Olofsson and Gottfried, 2015). Humans as a species appear, however, surprisingly good at detecting and discriminating odors, although they struggle to identify them, especially in decontextualized laboratory experiments studying participants pertaining to WEIRD (western, educated, industrialized, rich, and democratic) and ODD (old, deodorized, and desensitized) societies (Henrich et al., 2010; Roberts et al., 2020). However, ethnographic and ethnolinguistic studies report elaborated ways to conceptualize and lexicalize everyday odor impressions in traditional societies, including those related to the human body, (e.g., Majid and Burenhult, 2014; Barkat-defradas and Motte-florac, 2016; Majid et al., 2018), showing that the human sense of smell is far from being muted, especially in cultures where it bears everyday survival value (Majid and Kruspe, 2018; Majid, 2021). Nevertheless, the olfaction-language conundrum remains persistent among experimentalists, inducing numerous laboratory studies that think to circumvent it in relying on implicit measurements (e.g., brain or autonomous nervous system activity), especially since individuals typically respond to odors without awareness (Pause et al., 1998; Pause, 2012), and/or in following indirect approached, for instance examining how olfaction impacts other sensory modalities, such as vision.

Various levels of social information can be conveyed through body odors, including *individuality* (Hold and Schleidt, 1977; Mallet and Schaal, 1998; Platek et al., 2001; Lenochova and Havlicek, 2008), *self* (Hold and Schleidt, 1977; Lord and Kasprzak, 1989; Mallet and Schaal, 1998; Platek et al., 2001), *kin* (Porter and Moore, 1981; Porter et al., 1983, 1985, 1986; Porter, 1998; Schaal and Marlier, 1998; Weisfeld et al., 2003; Lundström et al., 2009; Schäfer et al., 2020), *age* (Haze et al., 2001; Yamazaki et al., 2010; Mitro et al., 2012), *sex* (Russell, 1976; Doty et al., 1978; Schleidt, 1980), and *personality* (McBurney et al., 1976; Sorokowska et al., 2012, 2016; Sorokowska, 2013a,b). Body odor can also inform about transient states such as *illness* (Moshkin et al., 2012; Olsson et al., 2014; Newman and Buesching, 2019), and *emotions* (e.g., Chen and Haviland-Jones, 1999, 2000; Prehn et al., 2006; de Groot et al., 2012, 2020; Zheng et al., 2018; for reviews, see Pause, 2012; Calvi et al., 2020; Kontaris et al., 2020). Beyond the mere consideration of the ability of olfaction to cue different levels of social information, several questions emerge on the potential interactions between the sensory channels processing this social information.

To date, the influences of vision on olfaction have been extensively investigated, with for instance the report of a facilitated perception of odors presented simultaneously with semantically congruent objects (Gottfried and Dolan, 2003), or the striking result of oenology students providing an olfactory description of white wine artificially colored red (with an odorless dye) as a red wine (Morrot et al., 2001; for other studies linking color and olfactory perception see Zellner et al., 1991; Österbauer et al., 2005). Overall, these vision-to-olfaction influences were mostly studied in relation to the food domain (for a review, see Verhagen and Engelen, 2006). By contrast, the converse direction of modulation - visual perception by olfactory stimuli - is only beginning to be scrutinized (e.g., Kuang and Zhang, 2014). Olfaction may however prove especially useful to hone our interpretation of social interactions. Real-life facial cues are often ambiguous, and may hide or fake true intentions and feelings, especially compared with typical lab settings endorsing the use of stereotypical-posed faces (Aviezer et al., 2017; Barrett et al., 2019). Natural body odor cues are more difficult for an individual to manipulate than facial cues, and even though one may know what facial movements are expected in a particular context, these olfactory cues are however involuntarily emitted, actually sometimes unbeknownst to the individual himself. In sum, olfactory-visual face perception may assuage the consequences of social uncertainty. In the following subsections, we present studies examining the influence of human body odors on different aspects of face processing, from the perception of invariant aspect of faces (e.g., identity, sex, and attractiveness) to the processing of transient facial expression of emotions (for a summary of included papers see Supplementary Tables 1–4).

## The Influence of Body Odors on the Processing of Invariant Face Characteristics

What makes a face attractive has been widely analyzed, and several determinants have now been identified including (but not limited to) symmetry, sexual dimorphism, averageness, and recently, facial adiposity and color (for reviews see Rhodes, 2006; Little et al., 2011; de Jager et al., 2018; Thorstenson, 2018). Under an evolutionary account that conceives facial attractiveness as an adaptation to solve the problem of mate choice, these components may serve as indicators of mate quality, such as health, resistance to parasites, or fertility (Thornhill and Gangestad, 1993; Little et al., 2011; Foo et al., 2017). However, and despite the popular maxim that “beauty is in the eye of the beholder,” face and person attractiveness is “more than meet the eyes” and is largely multimodal (Puts et al., 2012; Groyecka et al., 2017; but see Roth et al., 2021). With regard to mate selection, multisensory cues may well provide a better overall indication of mate quality than unisensory information. Indeed, even though one of these cues might not “honestly” advertises mate value (Zahavi, 1975), the simultaneous occurrence of fake quality cues on different sensory channels is unlikely (Rikowski and Grammer, 1999) – although nowadays that is why perfume and make-up are used (see Gaby and Zayas, 2017). Body odors may convey such a honest information as their saliency has been highlighted in mate selection (Havlicek et al., 2008; Lübke and Pause, 2015; White and Cunningham, 2017), and in the establishment of romantic relationships (for review, see Mahmut and Croy, 2019). Body odor pleasantness and facial attractiveness correlate weakly but significantly in both men (axillary odor: Roberts et al., 2011; Carrito et al., 2017; Mahmut et al., 2019; whole torso odor conveyed on a t-shirt: Gangestad and Thornhill, 1998; Rikowski and Grammer, 1999; Thornhill and Gangestad, 1999) and women (whole torso odor: Rikowski and Grammer, 1999; Thornhill and Gangestad, 1999; Thornhill et al., 2003), even though when it comes to mate choice, women are reportedly more prone than men to rely on olfaction (Herz and Cahill, 1997; Herz and Inzlicht, 2002; Havlicek et al., 2008; White and Cunningham, 2017; but see Foster, 2008).

Direct influence of body odors on the perception of face attractiveness has been reported, as implicit exposure to a male axillary odor increases attractiveness judgments of women toward male faces (Thorne et al., 2002), but not of males exposed to a female axillary odor and judging the attractiveness of female faces (Habel et al., 2021). The influence of body odors expands beyond attractiveness *per se*, following the “beautiful-is-good stereotype” (Dion et al., 1972; see also the “halo effect”; Nisbett and Wilson, 1977), according to which desirable traits are attributed to individuals considered as beautiful. Translated to olfactory issues, exposure to pleasant body odors (male axillary sweat) induced trustworthiness attribution in a Trust Game (Lobmaier et al., 2020), whereby participants gave more money to a trustee during the game. Similar findings were reported in women following exposure to an isolated component of human body effluvium (i.e., hexanal; van Nieuwenburg et al., 2019). Implicit exposure to hexanal not only affected interpersonal attribution of trust in a Trust Game, but also increased face trustworthiness ratings (van Nieuwenburg et al., 2019). The influence of the odorant on face trustworthiness ratings was particularly evident when presented outside of conscious awareness, thus ruling out an effect merely based on differences in odor intensity or pleasantness.

Recent findings have also highlighted the importance of the context and relationship status on these effects, as attractiveness judgments of highly attractive female faces decrease for pair-bonded, but not single men, implicitly exposed to the axillary odor of fertile (i.e., ovulating) women, interpreted as a defense against potential threats to the stability of their current relationship (Oren and Shamay-Tsoory, 2017). By contrast, women engaged in stable relationships showed a preference for the axillary odor of dominant men during their fertile phase, whereas single women did not (Havlicek et al., 2005; for similar results with face preference, see Little and Jones, 2012). Regarding dominance, although it is possible to make relatively accurate (i.e., above chance level) judgments of dominance based on body odor alone (worn t-shirt odor, Sorokowska et al., 2012; axillary odor, Sorokowska, 2013a,b; Sorokowska et al., 2016), the influence of body odor on the perception of facial dominance have not been formally examined so far.

Human body odors of various sources (e.g., axillae, skin, and breath) are discriminable by sex (e.g., Russell, 1976; Doty et al., 1978; Schleidt, 1980; Mutic et al., 2016), an ability thought to be mostly mediated by odor intensity (male axillary odors being rated as more intense than female axillary odors, Doty et al., 1978; Schleidt, 1980; Chen and Haviland-Jones, 1999), and fueled by the stereotypical overgeneralization that average men smell worse and more intensely than average women (Schleidt, 1980; Mutic et al., 2016; Carrito et al., 2017). Hence, body odors have been claimed to modulate face sex perception (Mutic et al., 2016). More precisely, androgynous faces (i.e., computer-generated faces constituted of equivalent female/male characteristic ratio) were rated as more feminine following exposure to a body odor (i.e., male or female axillary sweat) compared to a no-odor condition in female, but not male participants, which was interpreted as a femininity bias (Mutic et al., 2016). It is worth noting that both male and female participants displayed biased estimation of the androgynous faces in the absence of body odor, those faces being rated as slightly feminine and masculine by male and female participants, respectively. Thus, it could be considered that the body odor actually sharpened the perception of sex-ambiguous faces toward a more objective rating (i.e., closer to a sex-neutral estimation), rather than creating a true femininity bias.

Reminiscent of faces and fingerprints, body odors are idiosyncratic (Nicolaides, 1974; Penn et al., 2007), and their composition is largely under genetic control (Kuhn and Natsch, 2009; Natsch and Emter, 2020) producing a stable “odorprint,” albeit transiently modifiable, for instance through diet, emotion or hygiene (for review see Havlicek and Roberts, 2009). Even trained dogs are unable to discriminate between the body odors sampled on worn t-shirts of identical twins, as long as they follow the same diet (Hepper, 1988). This unique personal odorprint yields strong multisensory facilitation of self-face recognition (axillary odor, Platek et al., 2004), shortening reaction time for self-face recognition compared to other odors priming conditions (i.e., male or female axillary odor, androstenone, phenylethanol, or no odor), and suggesting that our own body odor is integral to the cognitive representation of our self. A similar multisensory facilitation would seem plausible for the processing of familiar individuals, such as relatives, mates, or friends, although the latter study did not find any priming effect of the body odor of participants’ co-workers/friends on the recognition of their face (Platek et al., 2004). However, it has been pointed out that these null findings likely stem from a lack of power due to the small samples (i.e., from 9 to 12 per experiment) used in the study rather than reflecting true absence of effect (Brédart, 2004). Being underpowered is especially problematic when comparing two conditions that may differ in effect sizes, and because we are “experts of ourselves” the magnitude of multisensory facilitation is likely much stronger for self-face recognition than for the recognition of other familiar faces (Brédart, 2004). A cautious conclusion would therefore be that the facilitation of self-face recognition through self-odor priming can be observed under conditions that, in contrast, do not allow facilitation of another familiar face recognition. For these reasons, it is actually unclear whether the influence of body odor on face identity recognition extends beyond self-face recognition and further investigations are needed.

## The Case of Androstenes

Among the large variety of compounds that constitute human body odor (de Lacy Costello et al., 2014; Ferdenzi et al., 2020), some androstenes present in axillary secretions received much attention in the last two decades, 5α-androst-16-en-3-one (androstenone), 5α-androst-16-en-3α-ol (androstenol), 4,16-androstadien-3-one (androstadienone), and to a lesser extent, estra-1,3,5(10),16-tetraen-3-ol (estratetraenol) (for a comprehensive review, see Havlicek et al., 2010). This focus is based on a widespread – although highly controversial – view that these compounds could constitute human sex pheromones (Rikowski and Grammer, 1999; Thornhill and Gangestad, 1999; Savic et al., 2001, 2005; Berglund et al., 2006, 2008; Zhou et al., 2014; Ye et al., 2019; Chen et al., 2021; for in-depth discussions of why these molecules should not be designated as pheromones, see Wysocki and Preti, 2004; Doty, 2010; Wyatt, 2015). Resolving this issue is beyond the scope of the current review, and we will hereafter follow a cautious approach considering androstenes as body odorants rather than “putative human pheromones”.

### Face Sex Perception

The production of androstenes is sexually dimorphic, with women emitting fewer quantities of androstenone than men (Gower and Ruparelia, 1993), and estratetraenol being mainly produced by pregnant women (Thysen et al., 1968). For this reason, androstenone (along with androstadienone and androstenol) was typically associated with males, and estratetraenol with females (e.g., Kovács et al., 2004; Zhou et al., 2014; Chen et al., 2021). These compounds were furthermore thought to convey information about masculinity and femininity, and to facilitate face sex perception in a multisensory context (Kovács et al., 2004). Initial findings in a sample of male participants have shown that androstadienone facilitated the perception of masculinity in female-to-male morphed faces (i.e., computer-generated linear continuum of faces with varying female/male ratio), while estratetraenol has no influence on the perception of facial femininity (Kovács et al., 2004). However, subsequent studies failed to replicate this effect, in both male and female participants (Ferdenzi et al., 2016; Hare et al., 2017), casting doubt on the potency of these compounds to influence face sex perception (Wyatt, 2015; Hare et al., 2017).

### Mate Selection and Attractiveness

Androstenes were further proposed to be related to mate selection and sexual behavior (Gower and Ruparelia, 1993; Thornhill and Gangestad, 1999; Savic et al., 2001), for instance by modulating judgments of attractiveness in the face of potential mates (e.g., Saxton et al., 2008; Niu and Zheng, 2020). Supporting this view, a number of studies reported that exposure to androstenes increase perceived attractiveness in faces (i.e., androstadienone: Saxton et al., 2008; Ferdenzi et al., 2016; androstenol: Kirk-Smith et al., 1978; Filsinger et al., 1985). However, some studies reported an effect on same-sex faces only, for male participants rating male faces (androstenol: Filsinger et al., 1985), whereas others reported an effect for opposite-sex faces, for female participants (fertile woman) rating male faces (androstadienone: Saxton et al., 2008; Ferdenzi et al., 2016), or for ratings of female but not male faces by both male and female participants (androstenol: Kirk-Smith et al., 1978). By contrast, other studies failed to find any effect on facial attractiveness judgments (i.e., androstenol: Black and Biron, 1982; androstadienone and estratetraenol: Hare et al., 2017; androstadienone: Lundström and Olsson, 2005, see also Frey et al., 2012) or even found decreased attractiveness ratings following exposure to the compounds (androstenone: Filsinger et al., 1985; Kirk-Smith and Booth, 1990; androstadienone: Parma et al., 2012). In sum, the evidence is mixed as to whether androstenes reliably influence the appraisal of attractiveness in faces. However, before drawing any conclusions regarding the evolutionary role of androstenes in mate choice scenarios, it is important to note that the effects of these compounds are highly context-dependent (e.g., of the sex of the experimenter, Jacob et al., 2001; Lundström and Olsson, 2005), and might need to be embedded within a relevant social context, including for instance realistic male–female interactions (e.g., real dating situations, Saxton et al., 2008).

### General Social Cognition

Another possibility is that, beyond mate selection, androstenes plays a role in more general aspects of social cognition, such as person perception. Behavioral evidence supports this view, as indicated by reports of increased generosity in female, but not male, participants (Perrotta et al., 2016), or increased cooperation in male participants (Huoviala and Rantala, 2013) following exposure to androstadienone during economic games (i.e., dictator game: Kahneman et al., 1986). More directly related to face processing, exposure to androstadienone fastens reaction speed toward schematic angry faces compared to happy faces (Frey et al., 2012), but also increases the judgment of dominance in faces (Banner and Shamay-tsoory, 2018), and triggers gaze avoidance (Banner et al., 2019) in men with high social anxiety. These two latter results however suggest that the signaling properties of androstadienone, if any, are very subtle as the effects were only observed for men with social anxiety, a population known for being hypersensitive to non-verbal social cues (Gilboa-Schechtman and Shachar-Lavie, 2013). Furthermore, a visual priming study using neutral faces reported no influence of androstadienone on attentional prioritization of social over non-social stimuli (see study 3 in Hummer and McClintock, 2009). Besides, several studies using the modified emotional Stroop task (e.g., faces with fearful and happy expressions presented with the words “happy” or “fear” written across them, Etkin et al., 2006) either found small effects on reduction of error rates, but not reaction times (Hornung et al., 2018b), reported findings limited to angry faces and to male participants (Hornung et al., 2017), or failed to find effects of androstadienone (Hornung et al., 2018a).

### Androstenes, Body Odors, and Face Perception

Overall, it appears that many results about the effects of androstenes on face perception are fairly contentious, with multiple research groups reporting conflicting findings. The very choice to specifically investigate the signaling value of androstenes among the myriad of other constituents of human body odors (e.g., de Lacy Costello et al., 2014) have been found somewhat arbitrary, and their biological relevance is yet to be demonstrated (Havlicek et al., 2010; Wyatt, 2015; Hare et al., 2017). Moreover, their use in studies at physiologically implausible concentrations (e.g., for concentrations of several order of magnitude above natural quantities, see Savic et al., 2001, 2005; Berglund et al., 2006), dampened the strength of the conclusions regarding their physiological and behavioral effects. Thus, provided that the focus is put on cognitive mechanisms or effects rather than on the molecules, the use of axillary extracts in their entirety seems a safe way to cope with these pitfalls (Wyatt, 2015).

## The Influence of Body Odor on the Perception of Transient Face Characteristics

Aside from conveying invariant social information, body odors also communicate more dynamic information related to emotional states, and humans have been found able to discriminate such “emotional body odors” (e.g., Chen and Haviland-Jones, 2000). Humans reliably discriminate between body odors collected on participants who have experienced events that induce fear or happy states (Ackerl et al., 2002; Zhou and Chen, 2011; Haviland-Jones et al., 2016a). These observations laid the ground for research on the chemical communication of emotions in humans, with a particular focus on fear/stress chemosensory cues (de Groot and Smeets, 2017; reviewed in Schaal, 2013) probably in relation with similar processes noted since decades in other vertebrates (e.g., Verheggen et al., 2010).

Anxiety odor (i.e., collected from the axillae just before an academic examination in male students) was found to disrupt the emotional priming of a happy face on a neutral face in a two-alternative forced choice (2AFC) task (Pause et al., 2004). In typical unisensory (i.e., visual) priming paradigms (Murphy and Zajonc, 1993; Cameron et al., 2012), neutral target faces tend to be found more pleasant when primed by happy compared to fear or sad faces. Exposure to an anxiety odor thus decreased the pleasantness of neutral faces primed by happy faces, although in female participants only. By contrast, exposure to a control body odor (i.e., collected during mere physical exercise) did not affect the visual priming, as neutral faces were judged more positively when primed by happy faces rather than by negative facial expressions (Pause et al., 2004). From these results, it is difficult to determine whether the anxiety odor decreased the positive value of the happy faces, of the neutral faces, or both. In any case, these findings indicate that anxiety odor cues can alter the perceived valence of expressive faces. These results were however limited to judgment of neutral faces primed by happy faces, as priming with fearful or sad faces did not make neutral faces more unpleasant following exposure to anxiety odor, presumably because participants’ response reached ceiling (Pause et al., 2004). Tentative interpretations were a dominance of chemosensory stimuli when sensory channels convey incongruent social cues, or a processing priority of anxiety-related information over positive and neutral information (for experimental support of the latter view, see de Groot et al., 2014b).

Further evidence that emotional chemosensory cues can influence face processing were provided by a psychophysical study showing that exposure to fear body odor (collected in axillary sweat after watching a 20-min clip excerpted from horror movies) biased female participants toward interpreting ambiguous fear-to-happy morphed facial expressions as more fearful in a 2-AFC task (Zhou and Chen, 2009). These findings indicate that visual and olfactory stimuli were integrated in an emotion-congruent manner (i.e., based on the redundant fear information), resulting in a face percept biased toward fear. Similar findings were recently reported using fear-to-disgust morphed faces, with “disgust” faces being perceived as more “fearful” by female participants after sniffing fear axillary sweat (de Groot et al., 2021). Fear axillary odor was also found to shorten the time interval that fear faces, but not disgust or neutral faces, took to reach visual awareness using a breaking Continuous Flash Suppression technique (i.e., b-CFS, the dichoptic presentation of dynamic noise to the dominant eye and a target stimulus to the other, which momentarily suppresses the target stimulus from visual awareness), again in a sample of female participants (Silvia et al., 2020). The influence of axillary fear sweat on the perception of emotional faces extends to male participants, biasing ratings of a continuum of neutral-to-happy morphed faces as less happy (Zernecke et al., 2011), and biasing ratings of neutral-to-fearful morphed faces as more fearful (Wudarczyk et al., 2016), in the context of a fear odor compared to an exercise odor. It is worth mentioning that in the aforementioned studies the effect was mainly evident for the most emotionally ambiguous faces or during challenging stimulation parameters hampering visual perception (e.g., the b-CFS technique). This suggests that the relative effectiveness of olfaction to communicate emotion in a multisensory context increases to the extent that perception in the other sensory modality (here, vision) is ambiguous. This is in keeping with the basic multisensory integration principle of “inverse effectiveness” according to which multisensory integration is more effective when the single modalities evoke relatively weak responses when presented in isolation (Meredith and Stein, 1983). In some respects, these findings also relate to the multisensory “reliability” rule holding that more weight is given to the modality providing the more precise (or more appropriate, see Welch and Warren, 1980) perceptual estimate depending on the task at hand (Ernst and Bülthoff, 2004), following a statistical optimal scheme (i.e., Maximum Likelihood Estimation, see Ernst and Banks, 2002). Further supporting this view, fear axillary odor has been found to quicken the recognition of fearful faces (but not other negative-valenced facial expressions such as anger and disgust) during the presentation of a stream of blurred face images gradually becoming clearer (Kamiloğlu et al., 2018).

Adding yet another layer of complexity, receivers sometimes fail to display emotion-specificity in their response to chemosensory cues conveyed in emotional axillary sweat odor. For instance, fear-related odor cues from the axillae have been found to boost the classification speed of emotional faces, regardless of the emotion depicted (i.e., fear, disgust, happy, and neutral faces: de Groot et al., 2015b), and to slightly increase the classification accuracy of dynamic facial expressions of both angry and happy faces (Rocha et al., 2018). Fear axillary odor also increased the readiness to detect both happy and fear faces using b-CFS (de Groot et al., 2018, but see Silvia et al., 2020). In a psychophysical task akin to that used in Zhou and Chen’s (2009), stress odors (i.e., collected from the axillae during skydiving) were found to sharpen visual discrimination of angry faces (Mujica-Parodi et al., 2009), refining the judgments of whether or not morphed faces (neutral-to-angry) were more neutral or more threatening. Thresholds of judgments between neutral and angry faces were similar for stress and control (physical exercise) odor conditions (i.e., the inflection points in the psychometric curves were similar for both odor conditions). In other words, the axillary stress odor increased accuracy in the assessment of angry and neutral faces rather than increasing the attribution of threat to neutral stimuli (Mujica-Parodi et al., 2009). The authors pointed out that an axillary stress odor may not be a consistent property to be associated with an angry face compared to a fear face, and might rather signal a nearby conspecific reacting to the angry individual (Mujica-Parodi et al., 2009). This odor cue may thus act as an alert signal increasing sensory vigilance overall (Mujica-Parodi et al., 2009; Rubin et al., 2012; de Groot et al., 2015b), inducing a general state of sensory acquisition (Susskind et al., 2008).

In fact, a number of studies have reported such increase in sensory vigilance following exposure to axillary stress odor (e.g., Prehn et al., 2006; de Groot et al., 2012; de Groot et al., 2015b). This phenomenon might partly account for the readiness to associate face and chemosensory stimuli in a non-emotionally specific manner in some studies (e.g., de Groot et al., 2015b, 2018; Rocha et al., 2018), even though the reasons for unsystematic increase of sensory vigilance are actually unclear. At the neural level, fear odor enhanced two early event-related-potentials (ERPs) components related to attention and face-processing (i.e., N1 and N170, respectively) during the presentation of anxious facial expressions (Adolph et al., 2013), consistent with an enhanced allocation of attention toward faces as expected under sensory vigilance. Note, however, that although the enhancement of N1 and N170 were observed when comparing a fear odor to a no odor condition, these ERP components were not different in amplitude when comparing the axillary fear odor to an exercise sweat condition (control odor), leaving open the question of whether the effect might stem from the exposure to any human body odor.

Such heightened vigilance effect seems restricted to fear/anxiety odor, as axillary sweat collected after other emotion-related challenges (happiness or disgust) have not been found to generate such an overall increase in vigilance (e.g., de Groot et al., 2018; Silvia et al., 2020). More generally, there is a paucity of data examining olfactory-visual interactions apart from fear/anxiety odors, among which results are mixed. Regarding the influence of a happy odor (collected from armpit), one study reported an increase of the unconscious readiness to detect happy faces in b-CFS (de Groot et al., 2018), whereas another study failed to find an influence of the happy odor on the perception of ambiguous fear-to-happy morphed facial expressions (Zhou and Chen, 2009). Disgust axillary odor (collected after participants watched disgusting movie excerpts) similarly failed to influence the processing of emotional faces (i.e., fear, disgust, and neutral faces) in b-CFS (Silvia et al., 2020). Using a non-emotional transient property, a study found that the axillary odor of sickness (i.e., an inflammatory response experimentally induced following injection of an endotoxin) negatively influenced the pleasantness ratings of faces of donors taken two hours after endotoxin (sick) or placebo (healthy) treatment (Regenbogen et al., 2017). The sickness odor decreased face pleasantness ratings compared to a control odor (i.e., an unused sampling pad), although not significantly when compared to the axillary odor of healthy individuals (Regenbogen et al., 2017; see also Sarolidou et al., 2020). Finally, although the most commonly studied carrier of human chemosensory cues is sweat, emotional tears (collected from female donors watching sad films) were also found to modulate face perception, whereby male participants rated female faces less sexually attractive after sniffing tears than after sniffing a saline solution (Gelstein et al., 2011, for discussion see, Gračanin et al., 2017; Sobel, 2017).

## Examining the Signaling Value of Emotional Body Odor: Competing Frameworks

Even though it stands to reason that emotional chemosensory cues do communicate information, these outcomes led to question the actual signaling value of emotional body odors (de Groot et al., 2015b). For instance, with regards to fear odor, an outstanding issue is whether the odor emulated the precise emotional state of the sender in the receiver (de Groot et al., 2012), or alternatively whether it generated a non-specific state of high arousal and negative valence unrelated to fear, upon which the fear emotion category is attributed depending on contextual factors (Hess and Fischer, 2013; Barrett, 2017). The former view relates to a widespread conception in psychology considering that emotions are psychologically and biologically basic, discrete, and shared within and across cultures (Ekman and Friesen, 1971; Ekman, 1992a; Scollon et al., 2004; Izard, 2007; Ekman and Cordaro, 2011). Each emotional state is associated with a specific neurophysiological pattern (a definable brain circuit or affect program) producing particular expressive behaviors (e.g., facial expressions), and distinct autonomic correlates (Ekman, 1992a,b; Izard, 1993; Panksepp, 1998; Buck, 1999; Cacioppo et al., 2000; Cosmides and Tooby, 2000; LeDoux, 2000). According to this perspective, emotional chemosensory cues in senders should induce discrete emotions (de Groot et al., 2017), which should translate in receivers displaying emotion-specificity in their response to the chemosensory stimulation (e.g., Zhou and Chen, 2009; Zernecke et al., 2011; Kamiloğlu et al., 2018; de Groot et al., 2021).

However, reports that emotional odors can affect the perception of facial expressions beyond the emotion category of the chemosensory stimuli (e.g., Mujica-Parodi et al., 2009; de Groot et al., 2015b; Rocha et al., 2018) led to nuance the claim that emotional odors bear a one-to-one correspondence to specific emotions. These latter findings rather dovetail with an alternative framework holding that emotions are grounded in core affects, and originate from the integral blend of at least two fluctuating properties – valence and arousal – thought of as psychological primitives or building blocks of emotions (Russell, 2003; Barrett, 2006b, 2017; Wilson-Mendenhall et al., 2013; Siegel et al., 2018). In this view, emotions are not “natural kind” (Barrett, 2006a), but emerge from these core affects in association with bodily feelings, conceptual knowledge, prior experience, and expectations (Barrett, 2017). Regarding chemosensory perception, this questions what meaning is chemically communicated from the sender to the receiver, and whether the message provides enough clear-cut information (e.g., a “discrete emotion package,” or a chemosensory cue with some valence and arousal properties) to trigger a partial simulation of a previously experienced emotional episode in the receiver (de Groot et al., 2015b).

A recent integrative framework (de Groot et al., 2017) pointed out that a chemosensory cue can acquire signal property over time by associative learning, provided that some statistical regularities can be extracted from the chemical components carried by body odor emitted when individuals experience an emotional episode. For example, the emotional impact of androstenone is positively correlated with the amount of exposure to it during sexual experience (Knaapila et al., 2012), and recent evidence suggests that patterns of chemical volatiles emitted by the body correlate with emotional states (Smeets et al., 2020). If reliable associations can be shaped by learning chemical profiles and co-occurring circumstances, chemosensory cues will be able to communicate emotional information with behavioral consequences indistinguishable from what could be expected under a discrete account of emotion (de Groot et al., 2020). Therefore, after exposure to correlated input signals, the visual system could use olfactory signals to build more reliable percepts of a social scene (Kuang and Zhang, 2014). Such a perspective implies that our brains relies on statistical regularities from past experiences, suggesting that a developmental investigation of the early influence of olfaction on face perception would provide valuable insights on the origins of these olfaction-to-vision influences. Although limited, an emerging literature is beginning to unravel how human chemosensory stimuli influence, and even mediate, face perception during the first months of aerial life.

## Developmental Aspects: Olfaction-To-Vision Influences in Infancy

Olfaction is a sensory channel benefiting from an early start as nasal chemosensation is functionally operative from the periphery to the brain by the end of the second gestational trimester (Schaal, 1988; Schaal et al., 2004; Browne, 2008). The partial overlap between the chemical profile of colostrum/milk secretions and that of the amniotic fluid allows for an olfactorily-smoothed natal transition continuity between intra-uterine and extra-uterine life (Schaal et al., 2020), notably through the exposure to colostrum, mother’s breast and body odor (Schaal et al., 2004; Marlier and Schaal, 2005; Klaey-Tassone et al., 2020). Such chemosensory continuity is essential in the adaptive development of other mammalian neonates, and is thought of as laying down the foundation for optimal social-emotional growth and cognitive development in humans as well (Schaal et al., 2020). Supporting this view, exposure to the odor of mother’s breast increases eye-opening in newborns (Doucet et al., 2007), favoring the infants’ first visual investigations of the maternal face. At later age, exposure to maternal body odor (presented using t-shirts worn during prior 3 nights) drives 4-month-old infants to allocate their attention toward social (i.e., faces) over non-social (i.e., cars) pictures presented pairwise from a screen, and especially to increase their active scanning of the eye area of the face (Durand et al., 2013). Moreover, the mother’s body odor shapes the visual categorization of faces (but not cars) presented embedded in a fast 6-Hz visual stream of widely variable natural images, enhancing a face-selective neural response in 4-month-olds (Leleu et al., 2020; Rekow et al., 2020). In addition, when using visual stimuli that infants could hardly categorize as faces (i.e., objects with face like configurations eliciting face pareidolia in adults), exposure to mother’s body odor boosts a selective response to these objects over brain regions typically responding to human faces in 4-month-olds (Rekow et al., 2021b).

Beyond the generic response to faces *per se*, maternal body odor also influences the processing of facial information, as indicated by the fact that 4-month-olds engage more their attention to their mother’s face than a stranger’s face in the context of maternal odor than they do in the context of a control (unworn t-shirt) odor (Durand et al., 2020). Rather unexpectedly, this face–odor association was further found to be category-specific rather than individual-specific, since body odors of other postparturient women also bolstered the visual attention of 4-month-old infants to mother’s versus stranger’s faces (Durand et al., 2020). Because other studies demonstrated that infants can discriminate their mother’s body odor from that of a stranger at a few days to weeks of age (Macfarlane, 1975; Cernoch and Porter, 1985; Porter and Winberg, 1999), it is unlikely that this pattern of findings reflects a confusion between mother’s and stranger’s body odor. Although speculative, a possibility is that the psychobiological state of postpartum leads to the release of a common pattern of chemical compounds in the body odor of postparturient women, to which infants would have responded categorically. In any case, a more specific response to the mother’s body odor was reported in older infants during the processing of emotional facial expressions (Jessen, 2020). Compared to the body odor of another mother (or a control odor), the exposure to their own-mother’s body odor reduced the typical neural response for fear faces in 7-month-olds, suggesting that maternal body odor can impact emotional learning in infancy (Jessen, 2020), although the underpinning mechanisms remain unclear.

Overall, these findings suggest that human body odor (represented here exclusively by the global maternal odor, data based on paternal odor being so far virtually inexistent) is processed as a social cue from early on, enhancing or facilitating the perception of faces (e.g., Durand et al., 2013; Leleu et al., 2020; Rekow et al., 2021b). This is consistent with the suggestion that olfaction is scaffolding the infant cognitive development, and helps to cope with the relative immaturity of the visual system in infancy (Schaal and Durand, 2012; Schaal et al., 2020).

Underlying this view is that albeit immature, infants have a cognitive integrity of their own (Bjorklund, 1997). In an evolutionary, ecological conception of development, the cognitive ability of infants should not be considered poorer than those of the child or adult organisms, but as fully fit to their ontogenetic stage, allowing to respond adaptively to whatever challenges their ecological niche poses to them (Spear, 1984). In other words, infancy should not be seen as an unfinished form of adult functioning, and some particular maturational constraint may in fact afford the infant some temporary advantage (Altmann, 2002; Kawai et al., 2012; Bjorklund, 1997). From this perspective, the immaturity of the visual system might prove advantageous inasmuch as this would entail a stronger weighting of olfaction in early learning (building on multisensory integration rules), fostering associative learning between chemical profiles emitted in conspecifics’ body odors and social information provided through other sensory channels, at a time where building a social brain is critical for the organism (Atzil et al., 2018). If so, the younger they are infants might prove more prone to olfaction-vision associative learning, and the influence of olfaction on face processing might reveal negatively associated with the development of visual perceptual skills in infancy. Indirect evidence suggests such a decrease of the weight of olfaction in adulthood compared to early infancy, since in adults the implicit exposure of human body odor no longer enhances the neural categorization response of faces as it does in infants (Leleu et al., 2020; Rekow et al., 2021a). Interestingly, an effect of body odor is however found on the adults’ brain response when visual perception is made more challenging (i.e., by using ambiguous face-like objects as stimuli presented for brief durations with forward- and backward-masking, Rekow et al., 2021a).

## Final Considerations and Future Directions

Body odors have been found to modulate the visual processing of both invariant (i.e., sex, attractiveness, and identity), and transient (i.e., emotional expressions and health) face properties. Spontaneously processed as carrying social information (Pause, 2012), body odors easily bind with face-related visual cues in a multisensory percept (Cecchetto et al., 2020), and overall, social smells bear on how we look at faces. However, large discrepancies emerge across studies in the way chemosensory information is integrated with faces, ranging from the multisensory integration of what seems redundant properties (e.g., identity: Platek et al., 2004; emotion: Zhou and Chen, 2009; Kamiloğlu et al., 2018; de Groot et al., 2021) to a more loosely matched correspondence with a rather unspecific influence of body odors (e.g., sex: Mutic et al., 2016; emotion: Mujica-Parodi et al., 2009; de Groot et al., 2015b; Rocha et al., 2018; health: Regenbogen et al., 2017).

### Causes of Variations of the Body Odor Influence

The puzzling versatility of the influence of body odors on face perception probably results from a variety of causes and may partially reflect difficulties in controlling the chemosensory information conveyed by natural odorants emitted by the body. For instance, the collection of fear odor includes a wide diversity of methods that markedly differ (e.g., first-time tandem skydiving, high rope course, academic examination, watching film clips excerpted from horror movies), which probably induce emotional states of variable intensity and quality (Lübke and Pause, 2015), which in turn may have consequences on the intensity and/or the quality of released odor stimuli (e.g., de Groot et al., 2020). Extreme physiological and psychological stress resulting from skydiving or high rope course likely activate different autonomic responses than watching horror movies while sitting in an armchair, the former inducing a strong physiological arousal potentially mixing various emotions (e.g., fear, relief, joy, and disgust), whereas the latter may evoke more specific emotions but with a weaker intensity (Pause, 2012; Lübke and Pause, 2015). Consequently, the pattern of volatile compounds released in both cases may differ, as may their influence on visual cognition in receivers. Note that labeling all these emotional body odors interchangeably as fear, stress, or anxiety odors may thus be somehow arbitrary and confusing.

Another source of variation may stem from the fact that the multisensory integration of two co-occurring sensory stimulations is not mandatory, and the brain has to determine which cues are derived from the same event before integrating them (i.e., the “correspondence problem”; Ernst and Bülthoff, 2004). Multisensory integration is thus both parameter and situation dependent (Munoz and Blumstein, 2020); one input being possibly discarded if too discrepant with the other source, as the benefits of ignoring a stimulus sometimes outweigh integrating two stimuli (Munoz and Blumstein, 2012). Inferring causation from correlation might be less straightforward for olfactory-visual than audio-visual integration, because olfactory perception is neither temporally nor spatially structured like visual perception (e.g., Sela and Sobel, 2010). Consequently, the embedding context along with the task at hand might crucially affect whether and to what extent olfactory cues will bind with visual information, which may contribute to the relative inconsistency of the influence of body odors on face perception in the reviewed studies.

It should be noted, however, that these theoretical considerations only hold as long as research findings are reliable, which sadly may turn out to be a too strong assumption considering the “reproducibility crisis”, as fewer than half of the findings have been estimated reproducible (Open Science Collaboration, 2015), and chemosensory research is no exception (Wyatt, 2020; Syrjänen et al., 2021). Various threats to reproducible science have been put forward (e.g., p-hacking, analytical flexibility, low statistical power, publication bias, Munafò et al., 2017; Nelson et al., 2018), and placed in the context of a competition-based model of science that values sheer output over good research (e.g., base evaluation of researcher on impact factor to determine hiring or promotion, Moher et al., 2018). This situation probably contributed to the publication of false positives resulting in the overall disparate outcomes reviewed here. This rather bleak picture should not hide recent improvements in practices (i.e., the “Psychology’s Renaissance”, Nelson et al., 2018), since means to increase the integrity of our discipline like pre-registered studies are beginning to emerge (e.g., de Groot et al., 2018; Silvia et al., 2020; Roth et al., 2021), and data disclosure is now widely encouraged (Morey et al., 2016; Wyatt, 2020).

### Potential Developments in Emotional Body Odors Research

Notwithstanding aforementioned remarks, based on the current body of research, there is now little doubt that body odors can communicate emotional information (de Groot et al., 2017). Nevertheless, it appears that not all emotions are equal when it comes to the transmission ability of body odor, as indicated by the overwhelming preponderance of findings related to fear/anxiety/stress odors compared to other emotional body odors such as happy or disgust body odors. This bias in the literature may stem from the greater potency of fear odors to trigger behavioral responses compared to other emotional body odors (Silvia et al., 2020), since, from an evolutionary perspective, failure to detect fear cues might prove more detrimental than missing happy cues (Öhman, 2009; Zhou and Chen, 2009). On the other hand, this bias may also reflect a lack of research on other body odors (Haviland-Jones et al., 2016b), perhaps precisely because other emotional odors may have been considered as carrying less evolutionary salience than fear/threatening cues (de Groot et al., 2015a). These two possibilities are not mutually exclusive, and future studies should further examine to what extent emotional body odors – aside from fear, stress, or anxiety odors – hold the ability to influence social perception based on vision (or audition, or touch). Recent promising findings suggest that this area of research is beginning to be scrutinized, with emerging investigations of body odors related to aggression (Pause et al., 2020), sexual arousal (Wisman and Shrira, 2020), dominance/competition (Fialová et al., 2020), or happiness (Smeets et al., 2020). Another line of research could involve investigations on how anosmic individuals process facial cues in comparison to normosmic individuals in both visual only and olfactory-visual contexts. While anosmic individuals are at obvious disadvantage to integrate olfactory and visual cues into multisensory percepts, they have been found to exhibit a specific expertise in tracking low-intensity visual cues of disgust or fear in faces (but not of happiness, sadness, anger, or surprise), showing better accuracy than normosmic participants in classifying these two facial emotions in a unisensory visual context (Lemogne et al., 2015). Interestingly, the expertise of anosmic participants in decoding facial emotion was correlated with the duration of their anosmia. Being unable to predict hazards based on olfactory cues, anosmic individuals may have been induced to attend more frequently and more deeply to others’ facial reactions elicited by olfaction and in detecting, at low-expressive intensity, related facial actions that reflect sensing of dangerous/disgusting items and express withdrawal. This increased accuracy in detecting facial disgust/fear might help permanently anosmic individuals to level with normosmic participants in the visual recognition of some facial emotions. Although speculative, a possibility is that in cases of transient anosmia, such as those caused by viral infections (e.g., Kollndorfer et al., 2015; Lechien et al., 2020; Petrocelli et al., 2021), the recuperation of olfaction might increase the detection of facially-expressed disgust or fear in prior anosmics, who may then surpass normosmics in olfactory-visual contexts. This remains to be formally examined, however. Furthermore, a comprehensive social cognition framework should also account for the developmental aspects of the multisensory social processing, and mechanisms underpinning early chemosensory influences on face processing in infancy and childhood may be important to understand for this reason. So far, however, only a few studies investigated how body odors influence face perception in infancy, and none has yet examined the response of infants to emotional body odors. Similarly, the developmental trajectory of olfaction-to-vision body odor influences in childhood is largely unknown, thus calling for more research to address these gaps in knowledge.

### Methodological Considerations: Sex Differences

A somewhat insidious blind spot that develops in olfactory research is related to sex differences. Typically females were shown to outperform males, notably by showing lower olfactory thresholds (Brand and Millot, 2001; Sergeant, 2010; Stevenson, 2010; for a meta-analysis see Sorokowski et al., 2019). These olfactory differences combined with findings reporting that women are also slightly better in processing social-emotional information (Proverbio et al., 2008; Proverbio, 2017) logically led to the observed male-female asymmetry in processing emotional chemosensory stimuli (de Groot et al., 2014a). Hence, to maximize the likelihood of finding an effect, many studies tested only female receivers exposed to body odor from male senders (e.g., de Groot et al., 2015b, 2021), because men possess larger apocrine sweat glands producing more intense body odor and are not subject to menstrual fluctuations of emission (Chen and Haviland-Jones, 1999; Sergeant, 2010). The downside of such experimental setting is the questionable generalizability of the results to the male population of odor receivers on the one hand, and to the female population as odor emitters on the other hand. Moreover, the effect sizes of olfactory sex differences are notably small (Sorokowski et al., 2019), suggesting to qualify this female advantage. In addition, interindividual variability in emotional body odor detection may prove to have a larger influence than sex differences, at least for the perception of happy and fear body odors (Haviland-Jones et al., 2016a). Strikingly, analyses of individual differences in emotional body odor perception indicated that almost 18% of individuals fail to discriminate happy and fear axillary odors from each other, and from a sterile gauze pad (Haviland-Jones et al., 2016a). At first glance, this could question the validity and utility of these odors as chemosensory cues in natural social situations. However, it should be kept in mind that the explicit detection of an odorant is far from a prerequisite for assessing an influence on the behavior of the receivers (Pause, 2012), even though such influence is denied by the participants themselves (for discussions, see, Haviland-Jones and Wilson, 2008; Haviland-Jones et al., 2016b). For these reasons, inclusion of both male and female participants should be fostered, and ideally, similar sex-clustered sampling should also be considered for the collection of olfactory stimuli.

### Ecological Considerations: The Use of Artificial Fragrances

Although the focus of the current review was put on human body odors, the widespread use of artificial fragrances (e.g., Roberts et al., 2010), however, requires to acknowledge their influence on everyday social communication (Allen et al., 2019; Spence, 2021), and regarding this latter point in particular, the interaction between extraneous fragrances and natural body odors should be carefully examined. Perfumes for instance may be used to exalt some particular invariant components of body odor (e.g., masculinity/femininity, personality traits, see Allen et al., 2019, for review) or perhaps hide others (e.g., emotional or illness cues), which may have influences on both receiver and sender sides in modulating person and face perception (e.g., Roberts et al., 2009; for review see Spence, 2021; Syrjänen et al., 2021). The nature and extent of interactions between artificial fragrances and body odor components related to transient states such as emotion or illness, however, are relatively unexplored, as are their impacts on social cognition. Note that skin exposure to scented products goes well beyond perfumes (e.g., deodorants, creams, soaps, and shampoos), and their influence on social perception can probably be traced back even to very early parent-infant social interactions, during which the premises of conditioning and associative learning between body odor and artificial fragrance may have been established. A complete understanding of olfactory communication through body odors must extend to ecologically valid conditions, thus artificial fragrances should be incorporated in future research aiming to better understand how artificial fragrances and natural body odor cues intertwine, and modulate social perception.

### Conclusion

The overarching goal of all cognitive processes is the control of adaptive action (Semin et al., 2012), which requires a perceptual system that produces reliable and robust percepts to cope with the basic uncertainty of the environment, including its social component. To do so, all brains must face the ill-posed problem of perception: multiple overt actions or covert attitudes are often possible based on a same unisensory stimulation, which can even have a low signal-to-noise ratio. In this endeavor, relying on multisensory perception to disambiguate unisensory inputs is highly effective, by combining percept estimates across sensory channels using a weighting process proportional to the reliability of each modality (Ernst and Banks, 2002), and by biasing perception away from the least certain modalities. Accordingly, because olfaction may be seen as a “weak sense” compared to the “dominant” visual system in adult humans, olfaction-to-vision interactions are usually reported in situations where quality of visual signals is not optimal (e.g., in early development) or degraded (e.g., Zhou et al., 2010; Kuang and Zhang, 2014), thereby making the reliability of visual and olfactory estimates comparable. Overall, the findings reviewed here nonetheless indicate that human chemosensory stimuli can have a substantial influence on the visual processing of faces, impacting situations of visual ambiguity (e.g., Zhou and Chen, 2009; Zernecke et al., 2011; Kamiloğlu et al., 2018; de Groot et al., 2021), though not limited to them (e.g., de Groot et al., 2015b; Wudarczyk et al., 2016). This discrete but pervasive influence of olfaction on visual-social processing may appear surprising when considering that, in many cases, the visual perceptual estimates were not especially compromised. However, sensory uncertainty may not only be characterized by weak stimulus signal-to-noise ratio or intensity, but also by variation in the predictive value of the stimulus (Munoz and Blumstein, 2012). It has been argued that social cognition is intrinsically marked by uncertainty because thoughts and intentions are largely hidden (FeldmanHall and Shenhav, 2019), and even emotional facial displays are not without ambiguities (Barrett et al., 2019). Seen from this vantage, the typical imbalance between vision and olfaction sensory estimates may thus be reduced in the social context, hereby offering more weight to chemosensory signals. In other words, during social encounters in our uncertain world, the nose could sometimes turn out to be as reliable as the eyes for an adaptive behavior.

## Author Contributions

FD, NM, BS, and AL ensured literature monitoring. FD wrote the first draft of the manuscript. All authors revised critically, read, and approved the submitted version.

## Conflict of Interest

The authors declare that the research was conducted in the absence of any commercial or financial relationships that could be construed as a potential conflict of interest.

## Publisher’s Note

All claims expressed in this article are solely those of the authors and do not necessarily represent those of their affiliated organizations, or those of the publisher, the editors and the reviewers. Any product that may be evaluated in this article, or claim that may be made by its manufacturer, is not guaranteed or endorsed by the publisher.
