# Supplementary material for: Olfaction in the Multisensory Processing of Faces: A Narrative Review of the Influence of Human Body Odors
Source: Front Psychol. 2021 Oct 5;12:750944. doi: 10.3389/fpsyg.2021.750944 (PMC8523678; doi:10.3389/fpsyg.2021.750944)
Supplement: Supplementary file 1 [file Table_1.pdf]

**Table 1** *Chronological summary of research investigating behavioral effects of body odors influence on invariant face properties*

| Study | N[1]-Sex[2] | Face property | Olfactory stimuli | Experimental task and visual stimuli | Behavioral effects of odorants |
|---|---|---|---|---|---|
| Thorne et al. (2002) | 32 F[2] | Attractiveness | M axillary odor vs. no odor | Attractiveness ratings of pictures of M faces | ↗ attractiveness ratings |
| Platek et al. (2004) | 7 F, 4 M[2] | Identity | axillary odors (self, M or F familiar and unfamiliar odors), androstenone, phenylethanol, or no odor | Face recognition of self, familiar, and unfamiliar faces | Facilitation of self-face recognition by self-odor; No effect of familiar odors |
| Mutic et al. (2016) | 16 F, 15 M | Gender | M and F axillary odors, neutral odor (unused pad) | Gender ratings of composite faces varying in F/M characteristic ratio | Sharpening the perception of sex-ambiguous faces |
| Oren and Shamay-Tsoory (2017) | 42 M | Attractiveness | F axillary odors (in ovulation vs. in luteal phase) | Attractiveness ratings of pictures of F faces | ↘ attractiveness ratings for pair-bonded compared to single men. |
| van Nieuwenburg et al. (2019) Exp 2 | 32 F | Trusworthiness | Hexanal, hexanal masked by eugenol, eugenol | Trustworthiness ratings of computer-generated M faces varying in trustworthiness | ↗ trustworthiness ratings |
| Habel et al. (2021) | 18 M | Attractiveness | F axillary odors (ovulating or pregnant women), and neutral odor (unused pad) | Attractiveness ratings of pictures of F faces | No effect |

[1] Outliers removed; [2]M/F, male/female; ↗, increase; ↘, decrease.

**Table 2** *Chronological summary of research investigating behavioral effects of influence of androstenes on invariant face properties*

| **Study (androstene)** | **N[1]-Sex[2]** | **Face property** | **Olfactory stimuli** | **Experimental task and visual stimuli** | **Behavioral effects of odorants** |
|---|---|---|---|---|---|
| Kirk-Smith et al. (1978) | 11 F[2], 11 M[2] | Attractiveness | Androstenol | Attractiveness ratings of pictures of F and M faces | ↗ attractiveness ratings of F faces by M and F participants |
| Black and Biron (1982) | 35 F, 36 M | Attractiveness | Androstenol, no odor | Attractiveness ratings of real M and F faces | No effect |
| Filsinger et al. (1985) | 132 F, 122 M | Attractiveness | Androstenol, androstenone, no odor | Attractiveness ratings of pictures of F and M faces | Androstenol: ↗ attractiveness ratings of M faces by M participants; Androstenone: ↘ attractiveness ratings of M and F faces by F participants |
| Kirk-Smith and Booth (1990) | 8 F, 8 M | Attractiveness | Androstenone | Attractiveness ratings of pictures of F and M faces | ↘ attractiveness ratings |
| Kovács et al. (2004) | 62 M | Gender | Androstadienone, estratetraenol, and neutral odor (water) | Gender categorization of computer-generated faces with varying F/M ratio | Androstadienone : facilitated masculinity perception ; Estratetraenol: no effect |
| Lundström and Olsson (2005) | 37 F | Attractiveness | Androstadienone, clove oil | Attractiveness ratings of pictures of M faces | No effect |
| Saxton et al. (2008) | 54 F | Attractiveness | Androstadienone, clove oil | Attractiveness ratings of real M (speed-dating) | ↗ attractiveness ratings of M faces by F participants |
| Hummer and McClintock (2009) Exp 3 | 30 F, 20 M | Social cognition | Androstadienone, clove oil | 2-back task (recall pictures of M and F faces presented 2 trial ago) | No effect |
| Frey et al. (2012) Rating task | 29 F, 31 M | Attractiveness | Androstadienone, eugenol | Attractiveness ratings of cartoon faces with happy or angry facial expressions | No effect |
| Frey et al. (2012) Joystick task | 29 F, 31 M | Emotion | Androstadienone, eugenol | Approach–avoidance task with cartoon faces with happy or angry facial expressions | Fastens reaction speed for angry faces compared to happy faces |

[1] Outliers removed; [2]M/F, male/female; ↗, increase; ↘, decrease.

**Table 2** (continued)

| Study (androstene) | N[1]-Sex[2] | Face property | Olfactory stimuli | Experimental task and visual stimuli | Behavioral effects of odorants |
|---|---|---|---|---|---|
| Parma et al. (2012) Exp 2 | 103 F[2] | Attractiveness | Androstadienone, clove oil | Attractiveness ratings of pictures of F faces | ↘ attractiveness ratings in F faces by F in luteal phase |
| Ferdenzi et al. (2016) | 39 F, 40 M[2] | Gender | Androstadienone, clove oil | Gender ratings of pictures of F and M faces | No effect |
| Ferdenzi et al. (2016) | 39 F, 40 M | Attractiveness | Androstadienone, clove oil | Attractiveness ratings of pictures of F and M faces | ↗ attractiveness ratings of M faces by F participants |
| Hare et al. (2017) Exp 1 | 46 (M & F) | Gender | Androstadienone, estratetraenol, clove oil | gender categorization of computer-generated androgynous faces | No effect |
| Hare et al. (2017) Exp 2 | 51 F, 43 M | Attractiveness | Androstadienone, estratetraenol, clove oil | Attractiveness ratings of pictures of F and M faces | No effect |
| Hornung et al. (2017) | 29 F, 27 M | Social cognition | Androstadienone, musk oil | Emotional Stroop task (M and F faces with happy, angry or fearful expressions) | ↗ error rates for angry faces in M participants; no effect on reaction times |
| Hornung Kogler et al. (2018) | 49 F, 27 M | Social cognition | Androstadienone, musk oil | Emotional Stroop task (M and F faces with happy, angry or fearful expressions) | No effect |
| Hornung Noack et al. (2018) | 46 F, 27 M | Social cognition | Androstadienone, or musk oil | Emotional Stroop task (M and F faces with happy, angry or fearful expressions) | ↘ of error rates; no effect on reaction times |
| Banner and Shamay-tsoory (2018) | 64 M | Dominance | Androstadienone, or eugenol | Dominance ratings of M faces | ↗ dominance ratings in M with high social anxiety |
| Banner et al. (2019) | 52 M | Dominance | Androstadienone, eugenol | Free viewing of pictures of M faces varying in dominance | ↗ gaze avoidance for dominance faces in M with high social anxiety |

[1] Outliers removed; [2]M/F, male/female; ↗, increase; ↘, decrease.

**Table 3** *Chronological summary of research investigating behavioral effects of emotional body odors influence on face perception*

| Study | N[1]-Sex[2] | Emotion induction | Olfactory stimuli | Experimental task and visual stimuli | Behavioral effects of odorants |
|---|---|---|---|---|---|
| Pause et al. (2004) | 8 F[2], 8 M[2] | Academic examination | M axillary anxiety or exercise odors | Emotional priming of neutral faces with neutral happy, fear, sad faces | Anxiety odor ↘ the pleasantness of neutral faces primed by happy faces in F |
| Mujica-Parodi et al. (2009) | 5 F, 9 M | First-time skydiving | M axillary anxiety or exercise odors | 2-AFC task with Neutral-to-angry morphed faces | Anxiety odor ↗ accuracy in the assessment of angry and neutral faces |
| Zhou and Chen (2009) | 48 F | Watching clips | M axillary fear, happy, or neutral odor (unused pad) | 2-AFC task with fear-to-happy morphed faces | Fear odor: ambiguous fear/happy morphed faces found more fearful; happy odor: no effect |
| Gelstein et al. (2011) | 24 M | Watching clips | F tears, saline or irritant solutions | Sexual attractiveness rating of pictures of F faces | F faces found less sexually attractive |
| Zernecke et al. (2011) | 15 M | High rope course | M axillary anxiety or exercise odors | Emotional rating of neutral-to-happy morphed faces | Anxiety odor ↘ happiness rating of neutral/happy morphed faces |
| de Groot, Smeets, and Semin (2015) | 31 F | Trier Social Stress Task | M axillary fear, or neutral odors | Emotion categorization of M and F faces depicting fear, disgust, happy, and neutral expressions | Fear odor quicken categorization of all facial expressions |
| Wudarczyk et al. (2016) | 10 F, 14 M | Academic examination | M axillary anxiety or exercise odors | Emotional rating of neutral-to-fearful morphed faces | Anxiety odor ↗ fearful rating of neutral/fearful morphed faces |
| Kamiloğlu et al. (2018) | 24 F | Watching clips | M axillary fear, or neutral odors | Emotion categorization of M and F faces depicting fear, disgust, anger, and neutral expressions | Fear odor quicken categorization of fear faces only |
| Gračanin et al. (2017) | 75 M | Watching clips | F tears or saline solution | Sexual attractiveness rating of pictures of F faces | No effect |

[1] Outliers removed; [2]M/F, male/female; neutral odor, sweat collected while watching non-emotional clips; ↗, increase; ↘, decrease.

**Table 3** (continued)

| Study | N[1]-Sex[2] | Emotion induction | Olfactory stimuli | Experimental task and visual stimuli | Behavioral effects of odorants |
|---|---|---|---|---|---|
| Rocha et al. (2018) | 46 F[2] | Academic examination | M[2] axillary fear, or neutral odors | Emotion categorization of videos of neutral-to-happy or neutral-to-angry M and F faces | Anxiety odor ↗ categorization accuracy of angry and happy faces |
| de Groot et al. (2018) | 96 F | watching clips | M axillary fear, happy, or neutral odors | Breaking Continuous Flash Suppression using M and F faces depicting fear, happy, and neutral facial expressions | Fear odor ↗ the readiness to detect both happy and fear faces |
| Silvia et al. (2020) | 29 F | Watching clips | M axillary fear, disgust, or neutral odors | Breaking Continuous Flash Suppression using M and F faces depicting fear, disgust and neutral emotional expressions | Fear odor sped-up access to awareness to fear faces only; Disgust body odor had no effect |
| de Groot et al. (2021) | 31 F | Watching clips | M axillary fear or neutral odors | 2-AFC task with fear-to-disgust morphed faces | Ambiguous fear/disgust morphed faces found more fearful |

[1] Outliers removed; [2]M/F, male/female; neutral odor, sweat collected while watching non-emotional clips; ↗, increase; ↘, decrease

**Table 4** *Chronological summary of research investigating effects of body odor influence on face perception in infancy*

| Study | Age | N[1]-Sex[2] | Method of measure | Olfactory stimuli | Experimental task and visual stimuli | Effects of odorants |
|---|---|---|---|---|---|---|
| Durand et al. (2013) | 4-month-olds | 26 F[2], 22 M[2] | Eye-tracking | Maternal body odor | Free viewing of F faces and cars shown side-by-side | ↗ of looking time toward faces, especially the eyes area |
| Durand et al. (2020) | 4-month-olds | 23F, 25M | Eye-tracking | Own- and other-mother body odor | Free viewing of pictures of own- and other-mother shown side-by-side | Both odors: ↘ looking time toward the face of other mother |
| Leleu et al. (2020) | 4-month-olds | 6 F, 12 M | FPVS-EEG | Maternal body odor | Free viewing of stream of objects and M and F faces | ↗ visual categorization of faces |
| Jessen (2020) | 7-month-olds | 38 F, 38 M | EEG | Own- and other-mother body odor | Free viewing of pictures of fear and happy F faces | Maternal odor: ↘ the neural response for fear faces; Other-mother odor: no effect |
| Rekow et al. (2021) | 4-month-olds | 9 F, 11 M | FPVS-EEG | Maternal body odor | Free viewing of stream of objects and face-like objects | ↗ visual categorization of face-like object as faces |

[1] Outliers removed; [2]M/F, male/female; FPVS-EEG, Fast Periodic Visual Stimulation coupled with Electroencephalography; ↗, increase; ↘, decrease.
